# Testing the testing effect on prolific: when retrieval practice fails to boost learning

**DOI:** 10.3389/fpsyg.2026.1727423

**Published:** 2026-01-29

**Authors:** Kevin Sigayret, Jean-François Parmentier, Franck Silvestre

**Affiliations:** 1 IRIT, Université de Toulouse, CNRS, Toulouse INP, Université Toulouse Capitole, Toulouse, France; 2 IRIT, Université de Toulouse, CNRS, Toulouse INP, IPSA, Toulouse, France; 3 IRIT, Université de Toulouse, CNRS, Toulouse INP, Toulouse, France

**Keywords:** crowdsourced samples, educational psychology, prolific, test-enhanced learning, testing effect

## Abstract

The testing effect—whereby retrieving information through testing improves long-term retention more than restudying—is one of the most robust and well-documented phenomena in educational psychology. However, its replicability in online crowdsourced settings remains uncertain. In this paper, we report two preliminary experiments examining whether the testing effect can be detected in studies conducted on Prolific, a platform widely used for behavioral and educational research. Although not conceived as direct replications, both studies relied on validated learning materials and incorporated key methodological features known to enhance the testing effect, including delayed posttests, corrective feedback, and both factual and application-based outcome measures. In both experiments, participants were randomly assigned to a retrieval-practice (test) or restudy condition. No significant differences were found between groups at delayed test, despite multiple safeguards aimed at promoting participant engagement (e.g., prescreening filters, attention checks, fair compensation). These null findings suggest that investigating learning phenomena that require sustained cognitive engagement may be more challenging with crowdsource samples. We argue that the absence of the testing effect in our studies is unlikely to reflect a theoretical limitation of the effect itself, but rather highlights challenges inherent to online research environments. While platforms like Prolific offer valuable advantages in terms of speed and sample diversity, their constraints should be carefully considered when studying learning effects that depend on deep engagement with learning material.

## 1 Introduction

Crowdsourcing platforms such as Amazon Mechanical Turk and prolific are increasingly used in psychological and educational research due to their accessibility, cost-efficiency, and participant diversity (Paolacci et al., 2010; Keith et al., 2017). However, concerns remain regarding data quality—particularly in educational psychology, where sustained cognitive engagement is essential (Hauser et al., 2019; Cheung et al., 2017). One of the most well-established effects in educational psychology is the testing effect, whereby retrieving information through testing improves long-term retention more than restudying (Roediger and Karpicke, 2006; Rowland, 2014). Because of its robustness, the testing effect provides a useful case for examining whether established learning effects can be detected in online, crowdsourced samples under controlled conditions. In this paper, we investigate whether the testing effect could be detected in two experiments conducted on the prolific platform using established materials and procedures. We report two preliminary studies conducted as part of a broader project, which were not intended as direct replications but rather as preparatory work for more complex investigations. Both experiments employed validated instructional materials widely used in educational psychology (e.g., Mayer and Chandler, 2001; Pan et al., 2023). These studies preserved the core materials and retrieval principles, but differed from prior laboratory implementations in participant population and procedural constraints associated with online data collection. Our results provide insights into the conditions under which the testing effect may or may not be observed in the present crowdsourced samples.

Testing is not only a means of assessment but also a powerful learning event. Theoretical accounts highlight that retrieval practice activates mechanisms that strengthen memory traces and improve access to learned content over time (Roediger et al., 2009). Among these mechanisms, the retrieval effort hypothesis (Yang et al., 2021) posits that the cognitive effort required to retrieve information enhances encoding. The transfer-appropriate processing (Yang et al., 2021) framework further suggests that when the cognitive processes involved in initial encoding align with those required during retrieval, performance at retrieval improves. Finally, motivational and metacognitive perspectives emphasize that testing can foster engagement and self-monitoring, encouraging learners to identify knowledge gaps and consolidate understanding (Yang et al., 2021). Testing has also been shown to support better organization of knowledge, reduce interference from previously learned material, and provide meaningful feedback to students (Roediger et al., 2011). Although early explanations emphasized mere re-exposure to the material, research has shown that testing yields benefits beyond those provided by additional study opportunities (Agarwal et al., 2008). Corrective feedback further enhances these effects (Roediger et al., 2009) and the absence of feedback can sometimes be detrimental, increasing the risk of false recognitions in later tests—especially with multiple-choice formats (Roediger et al., 2009; Kang et al., 2007).

A series of meta-analyses have established the reliability and generalizability of the testing effect. Effect sizes range from moderate to large, with estimates between *d* = 0.55 and *d* = 0.88 (Phelps, 2012), *g* = 0.50 (Rowland, 2014), and *g* = 0.61 to *g* = 0.70 (Adesope et al., 2017). The effect tends to be stronger in higher education contexts and when corrective feedback or performance incentives are included (Phelps, 2012; Pan and Rickard, 2018). Yang et al. (2021), in a large-scale meta-analysis of studies conducted in real classrooms, confirmed that the effect is observable across educational levels and learning outcomes—ranging from factual knowledge to conceptual understanding and even problem-solving. While the strongest effects are observed on retention, positive though smaller effects have also been reported on transfer tasks (Pan and Rickard, 2018), with elaborated retrieval practice and congruence between training and final test formats acting as key moderators. The testing effect has been demonstrated across a wide range of retrieval formats, including multiple-choice questions, cued recall, and free recall tasks (Adesope et al., 2017; Rowland, 2014). The critical mechanism is the act of retrieval itself rather than the specific test format. Across studies, the testing effect remains one of the most replicated and impactful findings in cognitive and educational psychology.

Importantly, the testing effect is not invariant across learners, tasks, or contexts. Prior work indicates that its magnitude depends on factors such as learner characteristics, task demands, and the degree of effortful engagement during retrieval. For instance, retrieval practice is most effective when learners engage in effortful and successful retrieval, and when motivational and self-regulatory factors support sustained engagement (Butler et al., 2017; Schwerter et al., 2025). Moreover, evidence from laboratory studies suggests that testing effects—including both positive and negative variants—may fail to generalize across participant populations, even when procedures are held constant (Mulligan et al., 2018). These findings highlight that the replicability and generalizability of retrieval-based learning effects depend not only on materials and procedures, but also on who the learners are and how they engage with the task. Nevertheless, given the robust evidence for the testing effect in controlled settings, an important open question is whether this effect can be reliably detected when studies are conducted using crowdsourced participant platforms. Recent evidence further suggests that retrieval practice may fail to confer learning benefits when task demands and cognitive load are high, even in controlled laboratory settings. For example, Redifer et al. (2026) reported no testing advantage when undergraduate students studied a lengthy and conceptually demanding academic text, with higher cognitive load mediating the relationship between retrieval practice and final test performance, such that increased cognitive load was associated with poorer delayed recall.

Despite the increasing popularity of crowdsourcing platforms in behavioral and educational research, concerns persist regarding the reliability and validity of data collected through such means. Online participants are often self-selected and may differ from target populations in key demographic or cognitive characteristics (Keith et al., 2017; Cheung et al., 2017). Specific issues include inattentive responding, repeated exposure to similar experimental paradigms leading to reduced naivety, deceptive behavior, and environmental distractions (Hauser et al., 2019). Workers may misrepresent themselves to qualify for studies or multitask during participation, which can compromise data quality (Cheung et al., 2017). The presence of “professional participants” or “Super Turkers” also raises concerns about sample independence and familiarity with standard experimental manipulations (Keith et al., 2017). To address these limitations, researchers have developed a range of methodological safeguards. These include the use of prescreening filters and custom qualifications, high approval thresholds (typically ≥95%), attention and comprehension checks, indirect eligibility screening, and metadata verification (Paolacci et al., 2010; Hauser et al., 2019). Fair compensation has been shown to reduce attrition and improve engagement (Keith et al., 2017; Albert and Smilek, 2023) and transparent reporting of recruitment procedures and exclusion criteria remains essential (Cheung et al., 2017; Hauser et al., 2019).

Comparative studies have revealed meaningful differences across platforms. Prolific and CloudResearch users consistently outperform Amazon Mechanical Turk (MTurk), Qualtrics, and SONA users on key data quality indicators (Douglas et al., 2023). Prolific participants show higher attentiveness, longer and more appropriate completion times, fewer signs of disengagement, and greater response consistency (Albert and Smilek, 2023; Douglas et al., 2023). They also outperform MTurk participants in educational problem-solving tasks that require sustained effort and deliberate engagement (Wang et al., 2024). While MTurk workers are generally more experienced, they are also more prone to multitasking and careless responding, especially in cognitively demanding tasks (Cheung et al., 2017; Albert and Smilek, 2023). Combined with superior prescreening tools, transparent demographic targeting, and cost-efficiency (Douglas et al., 2023), these strengths make prolific particularly well-suited for research in educational psychology. To examine whether the testing effect can reliably be observed in crowdsourced samples, we conducted two experimental studies using the prolific platform. Although the two experiments differed in learning materials and procedures, both were designed to assess the potential of prolific for supporting robust educational effects. In the following sections, we present the methods and results of each experiment, followed by a general discussion of their implications for educational research using crowdsourced samples.

Despite extensive evidence for the testing effect in laboratory and classroom settings, comparatively little is known about the conditions under which this effect can be detected in crowdsourced samples, particularly in studies requiring sustained engagement with learning materials. The present research addresses this gap by using the testing effect as a theoretically well-established benchmark to probe the limits and affordances of online participant platforms in educational psychology. Theoretically, these studies contribute to ongoing discussions about the role of engagement and task demands in enabling retrieval-based learning. Methodologically, they provide an empirical assessment of whether commonly recommended data-quality safeguards are sufficient to support robust learning effects in crowdsourced environments.

## 2 Method

We conducted two experiments using prolific^1^ to recruit participants and the Gorilla Experiment Builder platform^2^ to design our questionnaires. The two experiments differed in participant recruitment, materials, and procedures.

### 2.1 Participants

A total of 67 participants completed Experiment 1 (M_age = 20.8 years, SD = 1.81; 39 women). Prescreening criteria ensured that all participants were located in the USA or the UK, were currently pursuing an undergraduate degree, were fluent in English, and were aged between 18 and 25.

A total of 129 participants completed Experiment 2 (M_age = 47.4 years, SD = 12.4; range = 22–78; 74 women). Different prescreening criteria were applied: all participants were located in the USA or the UK, were native English speakers, declared a personal income of over £60,000 a year and had an approval rate on prolific of over 95%. Participants reporting conditions likely to interfere with task completion (dyslexia, vision or hearing difficulties) were excluded.

Participants were compensated £9 per hour (excluding prolific fees) for both experiments, exceeding prolific’s recommended minimum at the time.

### 2.2 Material

The learning material used in Experiment 1 was adapted from Pan et al. (2023), which itself built upon previous studies. The material comprised two texts (each approximately 500 words long), one on expressionist art and the other on ancient Rome. In each text, 10 key terms were italicized and followed by a one-sentence definition. Participants were instructed to memorize each term along with its corresponding definition. For each text, Pan et al. (2023) designed 20 different multiple-choice questions (with four possible answers). Ten questions assessed memory of what the key terms meant (definition questions) and 10 questions required to mobilize what had been memorized in a new, concrete situation, rather than simply restoring information (application questions). This material was chosen because it has been used in prior research in educational psychology and allows for the assessment of both factual recall and application, domains in which the testing effect is expected to occur. The two texts and the corresponding questions are provided in the Supplementary materials.

Experiment 2 employed a learning material on lightning formation that has been widely used in prior studies in educational psychology (e.g., Mayer and Chandler, 2001; Mayer et al., 2003; Bender et al., 2021). This material consisted mainly of a video animation describing the causal chain responsible for the formation of lightning in the sky. The video was accompanied by an audio narration that explained the on-screen animations. The video contained 19 key ideas that participants were instructed to memorize. The video and accompanying narration were reproduced using contemporary tools, while maintaining the original timing and structure (see Supplementary material S3 for the full video). The video lasted 2 min and 26 s. Prior to viewing the video, participants completed a questionnaire assessing prior meteorological knowledge, consisting of eight items rated on an 11-point scale. This questionnaire was designed and used (among others) by Mayer and Chandler (2001) to retrospectively exclude participants scoring higher than six. The list of the 19 key ideas and the meteorological knowledge questionnaire are provided in Supplementary materials. Additionally, the first three paragraphs of the text on ancient Rome, used in Experiment 1, were also presented during a preliminary screening phase for Experiment 2. This initial exposure served to select a subsample for the main experiment based on predefined criteria.

### 2.3 Procedure

Our two experiments were divided into several phases. At each phase, participants received an information notice detailing study objectives, procedure, duration, rights, and data confidentiality. Participants had to give their informed consent at each phase. Several attention checks were used across the experiments to identify and exclude inattentive participants, following prolific’s best practice guidelines for data quality.

#### 2.3.1 Experiment 1

Experiment 1 was divided into two phases. In the first phase, participants were asked their age, gender and the discipline they were currently studying (demographic data). Then, they were asked if they had already studied ancient Rome or expressionism at university and were excluded from the study if they did. The remaining participants were randomly assigned to two groups. Participants in the first group studied the ancient Rome text in the control condition and the expressionism text in the testing condition. In the second group, the assignment was reversed. The order of texts was counterbalanced across participants. In the control condition, participants were asked to read the text carefully before being given a list of the 10 key terms with the corresponding definitions (re-exposure condition). In the experimental condition, participants were asked to read the text carefully before having to answer the 10 definition questions with corrective feedback (testing condition).

**Figure 1:**
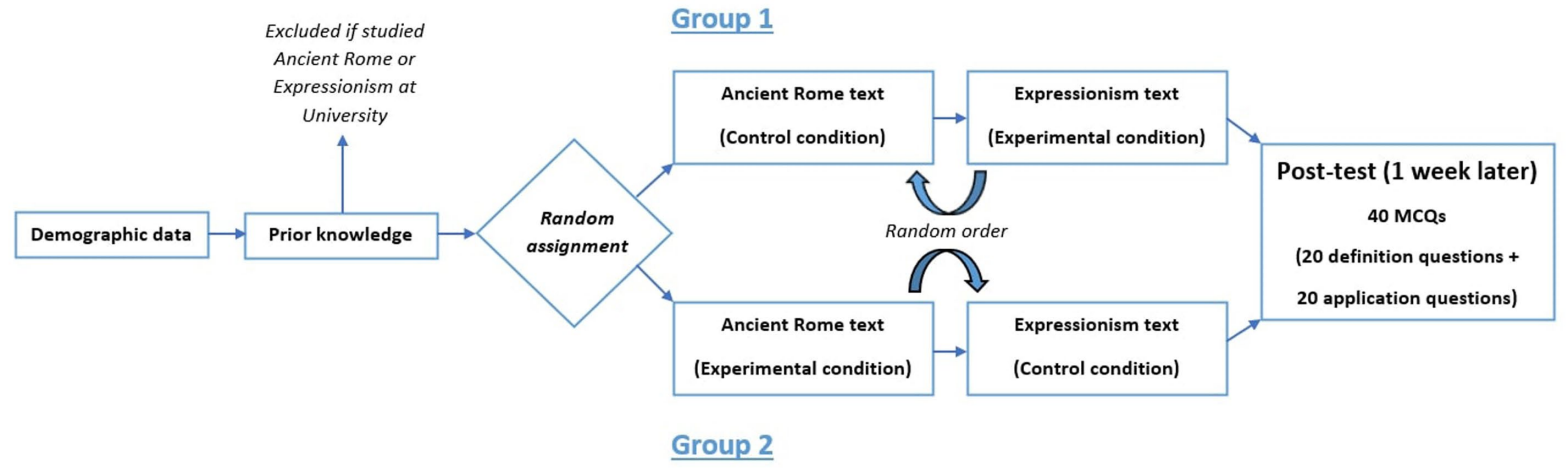
Design of experiment 1.

Participants who completed this first phase were invited to participate in the next follow-up second phase, 10 weeks later. In this second phase, participants had to answer the 40 multiple-choice questions (10 definition questions and 10 application questions for each text). Figure 1 shows the procedure for Experiment 1. In line with the testing effect, we expected higher delayed test performance in the experimental than in the control condition.

#### 2.3.2 Experiment 2

Experiment 2 was divided into three successive phases. In the first phase (preliminary screening phase), all participants were asked to carefully read a short expository text on ancient Rome, adapted from Experiment 1. They were then instructed to write a short summary of the text and explicitly told to spend at least 3 min on this free recall task. Completion times were monitored, and only participants who met the three-minute requirement were invited to take part in the subsequent phases.

The second phase began with an exclusion check: participants who reported having studied meteorology at the university level were immediately screened out. The remaining participants completed the prior meteorological knowledge questionnaire. Following the original procedure (Mayer and Chandler, 2001), participants who scored above six were excluded from the study. Eligible participants then proceeded to watch a short animated video on the formation of lightning, accompanied by an audio narration. The video could not be skipped or fast-forwarded, and participants were instructed to pay close attention and to memorize as much information as possible. After viewing the video, participants were randomly assigned to one of two groups. In the control group, participants watched the video a second time. In the experimental group, participants were asked to write down everything they could remember from the video. Consistent with prior work, retrieval practice was here implemented as a free recall task. This choice was motivated by meta-analytic evidence indicating larger testing effects with free recall than with recognition-based formats (Rowland, 2014). Following this phase, all participants—regardless of group—were provided with a written list of the 19 key ideas contained in the video. This step served as corrective feedback, ensuring that both groups had access to the complete set of target information prior to the delayed test. They were instructed to memorize these ideas carefully in preparation for the third and final phase.

**Figure 2:**
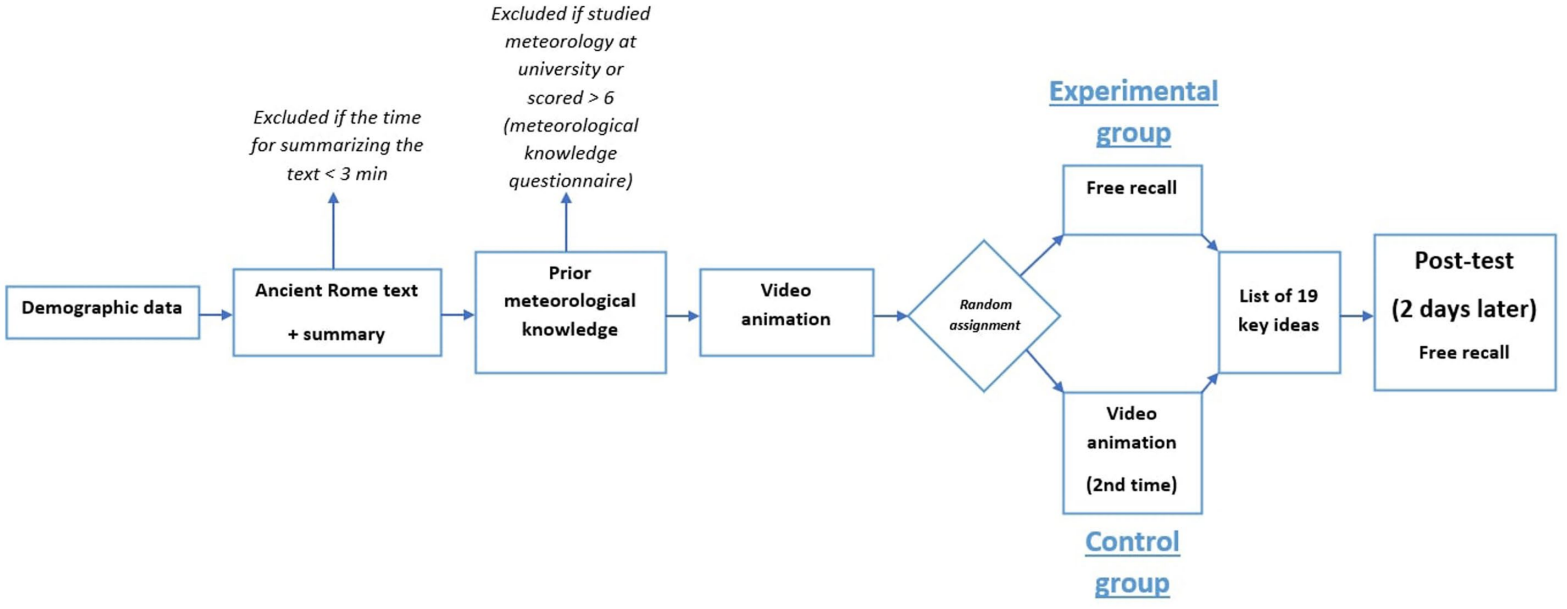
Design of experiment 2.

Two days later, all remaining participants were invited to complete the third phase. In this delayed recall phase, they were asked to write down as much information as they could remember about the process of lightning formation. Figure 2 shows the procedure for Experiment 2. In line with the testing effect, we expected higher delayed test performance in the experimental than in the control condition.

## 3 Results

Data were analyzed using Jamovi (v2.3.28). A linear mixed-effects model (LMM) was applied for Experiment 1 to account for repeated measures. In Experiment 2, due to non-normality, a Mann–Whitney *U* test was used.

### 3.1 Experiment 1

The most frequently reported fields of study among participants were Computer Science (*n* = 11), Psychology (*n* = 10), Business (*n* = 8), Engineering and Technology (*n* = 7), and Medicine and Health (*n* = 7). In the experimental condition, participants completed 10 multiple-choice training questions after reading the text. Training performance did not significantly differ between the Expressionism text (*M* = 6.34, SD = 2.30) and the Ancient Rome text (*M* = 6.58, SD = 2.45), t(65) = 0.41, *p* = 0.68.

Table 1 shows participants’ mean Definition, Application, and Total scores during phase 2, as a function of their learning condition (experimental or control) and the text they studied (Expressionism and Ancient Rome).

**Table 1:** Mean scores (SD) for definition, application, and total performance, by condition and text.

Text	Condition	Definition score (/10)	Application score (/10)	Total score (/20)	*N*
Expressionism	Control	4.50 (2.78)	4.18 (2.83)	8.68 (5.19)	38
	Experimental	5.21 (1.97)	3.66 (1.82)	8.88 (2.84)	29
Ancient Rome	Control	5.93 (2.42)	4.90 (2.18)	10.83 (3.70)	29
	Experimental	5.58 (2.43)	4.89 (2.48)	10.47 (4.42)	38

A total of 169 participants were initially recruited for phase 1. Of these, 42 were excluded upfront due to prior university-level coursework in Expressionism or Ancient Rome, and 7 did not complete phase 1 (due to failed attention checks or unknown reasons). Among the 120 remaining participants, only 67 (39.6%) completed both phases, resulting in substantial attrition. Forty-nine participants did not return for phase 2, and four failed attention checks.

Linear mixed-effects models were conducted to examine the effect of learning condition on post-test performance. The first LMM tested the effect of learning condition (experimental vs. control) on the total post-test score. The model included condition as a fixed effect and participant as a random intercept. No significant effect of learning condition on total score was found, *F*(1, 66) = 0.13, *p* = 0.72. Similar null effects were found for Definition score [*F*(1, 66) = 0.94, *p* = 0.33] and Application score [*F*(1, 66) = 0.27, *p* = 0.61]. Assumptions of normality were met based on inspection of model residuals (Shapiro–Wilk tests, *p* > 0.05 for all models).

The estimated completion time was 25 min for phase 1 and 20 min for phase 2. However, actual completion times were lower and showed substantial variability across participants (M = 13.7 min, SD = 9.02 min for phase 1; M = 15.4 min, SD = 10.8 min for phase 2). These averages were calculated for all participants who completed each respective phase, including those who did not take part in the subsequent phase.

Phase 1 cost approximately £600 (for 120 paid participants), and phase 2 approximately £270 (for 67 participants), including prolific fees.

### 3.2 Experiment 2

As in experiment 1, we observed high attrition. A total of 426 participants were recruited for the first preliminary screening phase, but only 129 (30.3%) completed the entire experiment. Of the 240 participants who passed the “3-min rule” for the text summary and were invited to phase 2, 27 did not participate in phase 2, 42 were excluded due to prior study of meteorology or high domain knowledge, 12 did not complete phase 2 (due to failed attention check or unknown reason), 21 did not participate in phase 3, and 9 did not complete phase 3 (same reasons).

Participants’ performance was assessed based on their ability to recall as many as possible of the 19 key ideas presented in the instructional video. In line with previous research (Mayer and Chandler, 2001; Mayer et al., 2003; Bender et al., 2021), the order in which ideas were recalled was not evaluated. Scoring was performed by a trained rater who was blind to the participants’ experimental condition. Each recalled idea was scored dichotomously (1 = idea present, 0 = idea absent), resulting in a total score out of 19. We compared the control group (M: 6.51; SD: 5.74) and the experimental group (M: 7.44; SD: 4.65) using a Mann Whitney U test. Results showed no significant difference between the groups (*U* = 1773; *p* = 0.17; *r* = 0.14)^3^.

The estimated completion time was 5 min for the first preliminary screening phase, 7 min for phase 2, and 8 min for phase 3. Actual completion times were close to expectations but showed substantial variability across participants (M = 10.2 min, SD = 5.7 min for phase 2; M = 7.1 min, SD = 3.9 min for phase 3).

Including prolific fees, preselection phase cost around £300 (for 426 paid participants), phase 1 cost around £175 (for 159 paid participants) and phase 2 cost around £155 (for 129 paid participants).

## 4 Discussion

Across two experiments conducted using the prolific platform, we failed to observe a statistically significant testing effect—despite using learning materials and procedures that have previously yielded robust effects in educational research. While our studies were initially designed as preliminary steps in the development of experimental materials, their failure to elicit the expected testing effect under the present conditions raises important questions about the reliability of crowdsourced data for research requiring sustained cognitive engagement.

In both experiments, we closely followed best practices from the testing effect literature: participants either restudied material or engaged in retrieval practice, with feedback provided and a delayed test phase introduced in both studies. These methodological conditions have been shown to amplify the testing effect (Roediger and Karpicke, 2006; Pan and Rickard, 2018). Moreover, the instructional materials used were derived from well-established materials (e.g., Mayer and Chandler, 2001; Pan et al., 2023), and the outcome measures targeted both factual recall and application, which are domains where testing effects typically emerge (Yang et al., 2021). Despite these safeguards, neither study showed a significant retrieval advantage. An *a priori* power analysis indicated that the sample size was sufficient to detect a medium-sized testing effect, as typically reported in the literature. In Experiment 1, performance was comparable across conditions. In Experiment 2, which implemented stricter inclusion criteria and procedural refinements (e.g., shorter phases, enforced viewing time), we observed numerically higher scores in the testing condition, but this difference failed to reach significance. These findings stand in contrast to decades of research highlighting the robustness and generalizability of the testing effect (Rowland, 2014; Adesope et al., 2017; Yang et al., 2021), and they prompt a closer examination of the potential limitations inherent to online data collection environments.

Our results suggest that, under the specific materials and procedures used here, detecting the testing effect in a crowdsourced sample may be more challenging than in contexts where learner engagement can be more closely monitored. This interpretation aligns with prior evidence showing that retrieval-based learning effects are contingent on effortful engagement and learner characteristics. Butler et al. (2017) demonstrated that retrieval promotes transfer primarily when retrieval attempts are effortful, whereas superficial engagement limits these benefits. Similarly, Schwerter et al. (2025) showed that even in authentic educational settings, practice testing is most effective—and most frequently used—by learners with sufficient motivation and self-regulatory resources. Differences in participant populations have also been shown to moderate the replicability of testing effects under otherwise similar procedures (Mulligan et al., 2018). In this light, our findings are best interpreted not as a failure of the testing effect per se, but as reflecting contextual and population-related constraints on its expression in crowdsourced environments.

Although we employed multiple methodological safeguards—including prescreening filters, English fluency, high approval rates, exclusion of participants with learning impairments, and fair compensation—several indicators pointed to possible suboptimal engagement. In Experiment 1, we observed extreme variability in completion times, with some participants completing the study unusually quickly, suggesting superficial task engagement. In Experiment 2, which included a preselection phase based on a minimum time requirement for a written recall task, 56% of participants failed to comply with the instructions, highlighting difficulties in enforcing adherence even with objective time constraints. We conducted exploratory analyses excluding participants with atypically short or long completion times, assuming rushed or multitasked behavior, but these exclusions did not alter the results. One possible explanation is that completion time constitutes an imprecise proxy for cognitive engagement in crowdsourced samples: extreme cases may reflect disengagement, but within the remaining range, variation in time-on-task does not necessarily map onto depth of processing. To address concerns about extrinsic motivation, Experiment 2 also restricted recruitment to individuals with an annual income above £60,000, under the assumption that these participants might be less driven by monetary incentives and more intrinsically motivated. This adjustment reduced access to undergraduate samples but was intended to improve data quality. Yet even under these stricter conditions, the expected testing effect did not materialize. While participants in the experimental condition outperformed those in the control group numerically, the difference was modest and statistically non-significant, raising questions about whether retrieval practice had any meaningful impact in this context.

These observations have broader implications for educational psychology research conducted via crowdsourcing. Learning studies often require participants to make an effortful investment in selecting, organizing, and integrating information (Mayer, 2014; Lei et al., 2018), processes that are sensitive to attentional fluctuations and motivational states (Seli et al., 2016). If participants approach these tasks with minimal engagement, the mechanisms underlying robust cognitive effects such as testing may fail to activate. This risk may be exacerbated on platforms like prolific, where participation is first-come, first-served and often driven by financial incentives. Participants may join studies indiscriminately and complete multiple tasks in rapid succession, which can reduce task-specific investment and increase cognitive fatigue or multitasking (Chmielewski and Kucker, 2020). Furthermore, crowdsourced studies do not offer the experimental control of laboratory settings, nor the ecological authenticity of classroom interventions. Instead, they occupy a hybrid space that blends convenience and scale with uncertainty and variability (Stewart et al., 2017). While crowdsourcing offers undeniable advantages for recruiting diverse samples and conducting relatively low-cost and large-scale studies, these benefits may come at the cost of reduced internal validity—particularly in research domains like education, where participant engagement is not incidental but central to the phenomenon under investigation.

Our findings must be interpreted in light of several limitations. First, despite careful prescreening, many of the inclusion criteria rely on self-report on prolific, which may be inaccurate or strategically manipulated. Second, the participant pool in Experiment 2, while more rigorously filtered, was no longer representative of the undergraduate population typically studied in testing effect research, limiting generalizability. Third, both experiments used a delayed post-test design and suffered from high attrition, which not only increased costs but also reduced statistical power and raised concerns about self-selection bias. Attrition is a well-documented challenge in crowdsourced research and varies widely as a function of task demands and study design. Prior work reports substantial variability in dropout rates on crowdsourcing platforms, ranging from minimal attrition in single-session studies to rates exceeding 50% in multi-phase designs with delayed follow-ups and cognitively demanding tasks (Hauser et al., 2019; Keith et al., 2017; Paolacci et al., 2010). In this context, the attrition observed in the present studies is consistent with patterns reported in the broader crowdsourcing literature. Fourth, in Experiment 2, recall performance was scored by a trained rater based on open-ended responses. Although the rater was blind to condition, scoring relied on subjective judgment about whether each of the 19 key ideas had been adequately expressed, introducing potential unreliability.

Despite these limitations, our findings do not invalidate the use of crowdsourced platforms for all types of research. Prolific may remain a viable option for studies involving simpler tasks, surveys, or reaction-time-based paradigms in which sustained engagement is typically less central. However, for cognitively demanding educational studies—particularly those involving extended learning phases, delayed assessments, and open-ended tasks—our results suggest that prolific samples may be less suitable, even under optimized methodological conditions. It is important to note that these findings should not be taken as evidence against the testing effect per se, which remains one of the most replicated phenomena in educational psychology. Rather, they highlight contextual limitations in eliciting this effect when participants are recruited via online platforms and tested outside of more controlled environments. These studies serve as a heuristic step toward designing more ecologically valid and methodologically robust investigations using crowdsourced samples. Future studies should aim to replicate these findings in controlled or ecologically valid contexts, such as university classrooms, where participant engagement can be monitored more closely and where the testing effect has repeatedly been demonstrated. An important consideration for future research concerns the trade-off between experimental control and ecological validity. While the use of generic, study-independent materials is well suited for isolating cognitive mechanisms, educational psychology ultimately aims to inform instructional practice. From this perspective, testing effects may be more meaningfully examined using curriculum-aligned materials embedded within authentic learning environments, such as university courses or digital learning platforms already used by teachers and students. Leveraging such platforms could allow researchers to study retrieval practice under conditions that better reflect real educational constraints while maintaining sufficient experimental control. Until then, researchers in educational psychology should be cautious when interpreting null effects from crowdsourced studies and consider the unique methodological challenges these environments pose.

## Data Availability

The datasets presented in this study can be found in online repositories. The names of the repository/repositories and accession number(s) can be found at: https://osf.io/qz8w9/?view_only=739131fcaf76421d9626901f9abc3e37.

## Footnotes

1
https://www.prolific.com/

2
https://gorilla.sc/

3 A descriptive inspection of recall patterns at the level of individual key ideas did not reveal any systematic differences between conditions.
