# Supplementary material for: Testing the testing effect on prolific: when retrieval practice fails to boost learning
Source: Front Psychol. 2026 Jan 29;17:1727423. doi: 10.3389/fpsyg.2026.1727423 (PMC12894256; doi:10.3389/fpsyg.2026.1727423)
Supplement: Supplementary file 1 [file Presentation_1.pdf]

Ancient Rome text (524 words; Flesch-Kincaid Level 15.0)

Life in the Roman Empire revolved around the city of Rome and its famous seven hills. Large Roman cities were resplendent with multiple theatres, gymnasiums, and baths. Roman residential architecture ranged from relatively modest domiciles to exquisite country villas. The capital city of Rome housed a plethora of elegant residences on Palatine Hill, the plateau on which the city was founded and the city's aristocratic quarter. The overwhelming majority of the population resided in insulas, which were tenements providing economically practical housing that were inhabited primarily by the laboring class. In contrast, the domus, which was a type of private, single-family residence of modest to palatial proportions, was inhabited primarily by the wealthy upper class.

The center of social structure in the Roman Empire was the family, which comprised a social, economic, and legal unit. The wife typically married into her husband's family and came under his legal authority (or that of his father if he was still alive), with her dowry integrated into the remainder of the estate under the ownership of the husband. The Paterfamilias was the oldest male and the head of the family, to whom his wife, his slaves, and possibly several generations of his descendants were subject and to whom title to all property was vested.

Associations between rich and poor individuals in the Roman Empire were structured by the bond existing between patron and client. The bond was exemplified via the routine practice of humble Romans partaking in the salutatio, which was the daily morning ritual of paying their respects in the houses of senators, who were obligated to protect them. These personal relationships conferred stability to the social hierarchy.

The head of state in Rome was the emperor. The emperor possessed authority by virtue of his tribunician proconsular powers. The tribunicia potestas vested in the emperor authority over Rome's civil government, including the power to preside over and to control the Senate, made him personally inviolable, and gave him the power to veto measures freely, summon the organs of government, and propose decrees and legislation. Further, the proconsular imperium gave the emperor authority over the Roman army, as well as the power to declare war, ratify treaties, negotiate with foreign leaders, and control senate membership.

The preeminent source of a Roman emperor's power and authority was the military. Roman soldiers swore the Sacramentum to the emperor, which was an oath of allegiance taken by soldiers to their commander that was sworn in a sacred place and using a formula that had a religious connotation. The emperor also had the allegiance and loyalty of the Praetorian Guard, which were household troops of Roman emperors that had significant political influence and generally participated in appointing emperors.

The death of an emperor often led to a crucial transition period. The senate was entitled to choose the new emperor, but most emperors selected a close family member as their successor. To ensure political stability, the new emperor had to seek a swift acknowledgement of his new status and authority. Many emperors thus paid a donativum, which was a donation given to each soldier upon the emperor's accession to secure their loyalty.

Ancient Rome: Key Terms and Concepts

1. Palatine Hill - plateau on which the city of Rome was founded and the city's aristocratic quarter

2. Insula - tenements providing economically practical housing and inhabited primarily by the laboring class

3. Domus - a private, single-family residence of modest to palatial proportions, was inhabited primarily by the wealthy upper class

4. Paterfamilias - the oldest male and the head of the family, to whom his wife, his slaves, and possibly several generations of his descendants were subject and to whom title to all property was vested

5. Salutatio - the daily morning ritual of paying respects in the houses of senators, who were obligated to protect their clients

6. Tribunicia Potestas - gave the emperor authority over Rome's civil government, including the power to preside over and to control the Senate, made him personally inviolable, and gave him the power to veto measures freely, summon the organs of government, and propose decrees and legislation

7. Proconsular Imperium - gave the emperor authority over the Roman army, as well as the power to declare war, ratify treaties, negotiate with foreign leaders, and control senate membership

8. Sacramentum - an oath of allegiance taken by soldiers to their commander that was sworn in a sacred place and using a formula having a religious connotation

9. Praetorian Guard - household troops of the Roman emperors that had significant political influence and generally participated in appointing emperors

10. Donativum - a donation given to each soldier on the emperor's accession to secure their loyalty

Ancient Rome: Definition MCQs

1. The Palatine Hill was a plateau...

a. on which the city of Rome was founded and the city's aristocratic quarter

b. that housed the majority of the population of Rome, who were mostly middle and working class

c. on which all religious assemblies in the city of Rome took place

d. on which Roman officials gathered for all governmental proceedings

2. An insula is a...

a. tenement providing economically practical housing and inhabited primarily by the laboring class

b. block of inexpensive housing meant for the financially struggling members of Roman society, such as widows, orphans, and beggars

c. collection of residential, commercial, and industrial buildings within the city of Rome designed to contain all basic living necessities

d. residential unit, similar to an apartment, meant to house members of the Roman army on leave

3. A domus is a...

a. type of private, single-family residence that was inhabited primarily by those in the wealthy upper class

b. single wing of the emperor’s palace that was inhabited by one side of his family

c. common architectural structure in Roman homes consisting of a large decorative beam held up by at least two pillars

d. type of small, single-family residence that was inhabited primarily by those in the middle and lower classes

4. The paterfamilias refers to...

a. the oldest male and the head of the family, to whom the title to all property was vested

b. all the males in a family, who bear the responsibility of caring for family members and ensuring their safety

c. the head of the household staff, usually male, in charge of the daily upkeep of the estate and delegating to the rest of the servants

d. the set of powers that grants full legal control over an estate, usually given to the most capable male family member

5. The salutatio is...

a. the daily morning ritual of paying respects in the houses of senators, who were obligated to protect their clients

b. the ritual prayer performed twice a day to the Roman gods in order to maintain health and prosperity

c. the daily convening of the staff with the head of the household to address upkeep and matters of the household

d. the daily greeting exchanged between family members and the head of the household

6. The tribunicia potestas...

a. gave the emperor authority over Rome's civil government, including the power to preside over and to control the Senate, to veto measures freely, and propose decrees and legislation

b. gave the Senate greater control over Rome’s civil government and the power to propose decrees and legislation

c. gave the emperor authority over diplomatic relations, including powers over trade and commerce, levying taxes, and arranging treaties with other states

d. gave the Senate greater authority over foreign and diplomatic relations, including powers over trade and commerce, levying taxes, and arranging treaties with other states

7. The proconsular imperium...

a. gave the emperor authority over the Roman army, as well as the power to declare war, ratify treaties, negotiate with foreign leaders, and control senate membership

b. gave the Senate some control over the Roman army, including the power to gather and redistribute troops, grant leave to soldiers and generals, and ratify treaties in wartime

c. gave the emperor greater authority over the judicial system of the Roman government, including the power to overturn trials and pardon people of capital punishment

d. gave the Senate greater authority over the judicial system of the Roman government, including the power to overturn trials and pardon people of capital punishment with a two-thirds majority vote

8. The sacramentum is...

a. an oath of allegiance taken by soldiers to their commander that was sworn in a sacred place and using a formula with a religious connotation

b. a ritual prayer performed by soldiers before battles to appease the gods and ask for good fortune in war

c. an oath of allegiance taken by senators to the emperor upon taking office

d. a ritual prayer performed by senators to the gods at the start of every senate convening to ask for wisdom and good fortune upon the state

9. The praetorian guard refers to...

a. the household troops of the Roman emperors that had significant political influence and generally participated in appointing emperors

b. the legion of troops assigned specifically to patrol and guard citizens of the capital city, Rome

c. mercenaries hired to assassinate any individuals who threatened Roman leadership

d. soldiers who were on reserve in the Roman army and called upon only when necessary in war time

10. A donativum is...

a. a payment given to each soldier on the emperor's accession to secure their loyalty

b. a monetary gift presented to Roman officials as bribery for political favors

c. a Roman emperor's monetary contribution to the state treasury upon their accession

d. an allowance granted to each member of the Senate at the start of their term

Ancient Rome: Application MCQs

1. Palatine Hill was NOT the location of...

a. the Temple of Magna Mater

b. the Tiber River bank trading post

c. Emperor Augustus' imperial palace

d. several senators' houses

2. Living in the insulas is characteristic of individuals at the ______ level of Roman society.

a. plebeian or working class

b. patrician or ruling class

c. equites or upper-class tradesmen

d. freedmen or former slave class

3. Flavia resided in Domus Augustus in the capital city of Rome. Which of the following best describes what her residence likely looked like?

a. A complex of homes featuring elaborate parks and gardens built atop terracing on a hillside.

b. A flat comprised of two rooms with a very simple design and without any running water.

c. A five-story apartment with shops and businesses on the ground floor and living quarters on the upper floors.

d. A house with a central atrium, gardens, a reception room, several dining rooms, and private bedrooms.

4. Faustus is a paterfamilias. Which of the following best describes his responsibilities?

a. hold some type of public office to prove good citizenship in the republic

b. provide food, clothing, shelter, and education for all members of his household

c. preside over a senate seat as a representative for his clients

d. hold religious ceremonies at least once a year to appease the household's patron god or goddess

5. While engaging in the salutatio, the patron Marcellus likely did which of the following?

a. saluted the emperor at a senate gathering

b. received his clients and heard their requests

c. greeted his officers at the start of a new military campaign

d. greeted fellow patrons and discussed current civil matters

6. Why might Emperor Tiberius have often been said to have shunned his tribunicia potestas powers?

a. He seemed to have wished for the Senate and the state to act without him.

b. He focused largely on tax reform during his reign rather than engage with diplomatic relations.

c. He made drastic changes to the legal structure of the empire.

d. He refused to grant any of his time to addressing military matters or campaigns.

7. Proconsular imperium was bestowed on Publius Cornelius Scipio Africanus by a vote of the people because...

a. he took temporary control of the Senate while the emperor was indisposed

b. he wanted to implement a popular prison reform plan

c. he volunteered to lead the second Roman expedition during the Second Punic War

d. with plebeian support, he wanted to propose a reduction in taxes for the working class

8. Which of the following is most likely the text used when swearing the sacramentum?

a. "I, [insert name], swear to act in accordance with the wellbeing of my people, to exercise the powers granted to me as emperor with restraint and wisdom, and to support and defend the Roman republic.”

b. "I, [insert name], swear that I shall command my soldiers with restraint and wisdom, that I shall never unduly put my people in the way of danger, and that I shall not seek to avoid death for the Roman republic.”

c. "The soldiers swear that they shall faithfully execute all that the Emperor commands, that they shall never desert the service, and that they shall not seek to avoid death for the Roman republic."

d. "The patrons swear that they shall act in accordance with the wellbeing of their clients, that they shall never unduly subvert the commands of the Emperor, and that they shall support and defend the Roman republic.”

9. Felix is a member of the Praetorian guard. Which of the following would best describe one of his privileges?

a. Vote in the senate

b. Private ownership of a plot of land

c. Help appoint the next emperor

d. Control over a portion of the state treasury

10. In 69 BC, Emperor Otho engaged in the practice of donativum. Which of the following best describes what that donativum likely entailed?

a. Engaging in various charitable acts that were highly publicized to the Roman citizenry.

b. Visiting the Imperial Senate and initiating discussions with Senators.

c. An official decree that declared authority over taxation policy across the Roman empire.

d. Gifts in the amount of one thousand and two-hundred fifty denarii, a form of Roman currency.

Expressionist Art Text (512 words; Flesch-Kincaid Level 15.7)

The Expressionist movement began primarily in Germany and Austria at the turn of the last century. Expressionism is an artistic style in which the artist seeks to depict not objective reality but rather the subjective emotions and responses that objects and events arouse. Expressionists’ goals were different from those of the preceding movement, Impressionism, which was an artistic style in which the artist attempts to accurately and objectively record visual reality in terms of transient effects of light and color.

Expressionists tended to use distortion, exaggeration, and fantasy in their work. Reflecting a common attitude of the time, they also perceived non-Western art as unevolved. Given that belief, their art also often demonstrated primitivism, which was an aesthetic idealization that aimed to recreate “primitive” experience by using nonindustrial elements that were meant to be closer to the origins of humanity and were consequently considered more pure. Many Expressionists also took inspiration from New Art, which was an international, middle-class

artistic movement that sought to reflect the intensive psychic and sensory stimuli of the modern city by using flat patterning and bold forms.

Within the early years of the Expressionist movement, two prominent groups emerged. Die Brücke was an organization of artists that were in revolt against what they saw as the superficial naturalism of academic Impressionism and who wanted to reinfuse German art with spiritual vigor through an elemental, highly personal and spontaneous expression. Their art often turned to simplified or distorted forms and often included unusually strong and unnatural colors in order to surprise the viewer and cause an emotional response. Blaue Reiter was a loosely knit organization of artists that used abstract forms and prismatic colors to explore the spiritual values of art as a counter to what they saw as the corruption and materialism of their age. The work of these artists was diverse, but it generally reflected an interest in free experimentation and spiritual expression. Unlike Die Brücke, their expressionism took the form of sensuous and romantic compositions.

Expressionism became a dominant style in Germany, during which it suited the post-war atmosphere of cynicism, alienation, and disillusionment. Some of the movement's later practitioners developed a style known as the Neue Sachlichkeit, in which artists created works executed in a more realistic style that reflected the resignation and cynicism of the post-World War I period in Germany. Some of these artists were also influenced by metaphysical painting, which was a style of painting using representational but incongruous imagery to produce disquieting effects on the viewer.

Metaphysical painting originated with the Italian artist Giorgio de Chirico, who took inspiration from the philosopher Friedrich Nietzsche. Nietzsche had argued that Greek tragedy arose out of the fusion of what he termed Apollonian and Dionysian elements. He used the term Apollonian to describe things relating to the god Apollo and representing reason, culture, harmony, and restraint. He used the term Dionysian to describe things relating to the god Dionysus and representing excess, irrationality, lack of discipline, and unbridled passion. Both of these elements are represented in metaphysical painting and some Neue Sachlichkeit works.

Expressionist Art: Key Terms and Concepts

1. Expressionism - artistic style in which the artist seeks to depict not objective reality, but rather the subjective emotions and responses that objects and events arouse

2. Impressionism - artistic style in which the artist attempts to accurately and objectively record visual reality in terms of transient effects of light and color

3. Primitivism - an aesthetic idealization that aimed to recreate "primitive" experience by using nonindustrial elements that were meant to be closer to the origins of humanity and was consequently considered more pure

4. New Art - an international, middle-class artistic movement that sought to reflect the intensive psychic and sensory stimuli of the modern city by using flat patterning and bold forms

5. Die Brücke - an organization of artists in revolt against what they saw as the superficial naturalism of academic Impressionism and who wanted to reinfuse German art with spiritual vigor through an elemental, highly personal and spontaneous expression

6. Blaue Reiter - a loosely knit organization of artists that used abstract forms and prismatic colors to explore the spiritual values of art as a counter to what they saw as the corruption and materialism of their age

7. Neue Sachlichkeit - artists who created works executed in a more realistic style that reflected the resignation and cynicism of the post-World War I period in Germany

8. Metaphysical painting - a style of painting using representational but incongruous imagery to produce disquieting effects on the viewer

9. Apollonian - relating to the god Apollo and representing reason, culture, harmony, and restraint

10. Dionysian - relating to the god Dionysus and representing excess, irrationality, lack of discipline, and unbridled passion

Expressionist Art: Definition MCQs

1. Expressionism is an artistic style in which the artist...

a. seeks to depict the subjective emotions and responses that objects and events arouse

b. seeks to depict the perceived emotions of both animate and inanimate objects

c. seeks to depict objective reality in terms of both light and color

d. seeks to depict the inner emotions of their human subjects using exaggerated facial features

2. Impressionism is an artistic style in which the artist...

a. attempts to accurately and objectively record visual reality

b. attempts to accurately and objectively record their own impression of their subjects

c. attempts to accurately and objectively record the general public's impression of different events

d. attempts to accurately and objectively record their first impressions of the objects and events they encounter

3. Primitivism was...

a. an aesthetic idealization that used nonindustrial elements meant to be closer to the origins of humanity and considered more pure

b. an aesthetic idealization that used only basic shapes to create unsophisticated depictions of reality

c. an aesthetic idealization that used mineral-based paints in natural colors to depict natural scenes

d. an aesthetic idealization that used industrial elements to depict scenes from nature

4. New Art an artistic movement that sought to...

a. reflect the intensive psychic and sensory stimuli of the modern city by using flat patterning and bold forms

b. break away from traditional forms by using industrial elements to depict even natural forms

c. reflect the rising industrial economy by using mostly straight lines and hard angles

d. depict the loud and chaotic sensory stimuli of the urban landscape by using primary colors and striking outlines

5. Die Brücke was an organization of artists...

a. who wanted to reinfuse German art with spiritual vigor through an elemental, highly personal and spontaneous expression

b. who wanted to depict objective reality with exact forms and naturalism that they found lacking in German art

c. who sought to bridge the gap between art and reality with a mixture of abstract and realistic elements

d. who countered the academic style of art at the time by exaggerating and distorting classical elements in color and shape

6. Blaue Reiter was an organization of artists...

a. that used abstract forms and prismatic colors to explore the spiritual values of art as a counter to the corruption and materialism of their age

b. that used fantasy elements to depict their desire to escape the harsh and unforgiving realities of the post-war age

c. that sought to reinfuse German art with hope and optimism to counter the disillusionment many young professionals faced at the time

d. that sought to represent metaphysical elements in opposition to the overly commercialized and utilitarian society of their time

7. Neue Sachlichkeit refers to...

a. artists who created works executed in a realistic style that reflected post-war resignation and cynicism

b. artists who sought to infuse hope in the post-war culture through an embellished and flamboyant style

c. artists who created works executed in a fundamental and bare style that reflected the stark and primitive nature of humanity at its core

d. artists who sought to accurately depict reality by translating what they saw as exactly as they could onto their work

8. Metaphysical painting is a style of painting that...

a. uses representational but incongruous imagery to produce disquieting effects on the viewer

b. uses distorted forms to create the impression of psychedelic and dream-like imagery

c. uses bright colors, concrete forms, and sharp contrasts to create a kaleidoscope effect

d. uses only symbolic imagery, drawing upon reality without trying to accurately depict it

9. The term Apollonian...

a. represents reason, culture, harmony, and restraint

b. represents gentility, character, and conformity

c. describes art that uses classical forms and academic realism

d. describes art that is conservative in style and promoted by an official academy

10. The term Dionysian...

a. represents excess, irrationality, lack of discipline, and unbridled passion

b. represents rebellion, deficiency, imbalance, and chaos

c. describes art that is exaggerated in style and includes fantasy elements

d. describes art that uses non-traditional and psychedelic elements

Expressionist Art: Application MCQs

1. Max Beckmann was often classified as an Expressionist artist but rejected the term. Which of the following could be a plausible reason Beckmann did not view himself as an Expressionist?

a. He objected to the academically correct depictions of Expressionist painters.

b. He objected to the strongly held traditions of Expressionism.

c. He was opposed to the introverted emotionalism of Expressionism.

d. He was opposed to the realism that was characteristic of Expressionist painters.

2. Which of the following methods would best help Impressionist painters achieve their aims?

a. Using patterns to symbolize different three-dimensional textures

b. Painting using only thin, transparent paint films (or glazes) to allow for layering that created distorted effects

c. Sketching quickly and without regard for exact accuracy to capture movement

d. Painting en plein air (or outdoors) in order to best capture the momentary effects of sunlight

3. Paul Gauguin often used primitivism in his art work. Which of the following likely describes his style and artistic goals?

a. Gauguin sought to escape European civilization and technology by including Tahitian motifs in his paintings and ceramics.

b. Gauguin sought to break from academic realism by using distortion and colorism in his work.

c. Gauguin used bold, intricate, and highly stylized patterning to evoke a sense of dissonance.

d. Gauguin used intense psychedelic imagery in an effort to escape the often intellectual and strict standards of his contemporaries.

4. A piece of furniture created in the style of the New Art movement would likely have...

a. Subtle and subdued curves and shapes

b. Rough or unfinished wood without varnish

c. Bulky and simple forms with clean lines

d. Bold whiplash (a highly stylized double curve) patterns

5. Which of the following would best describe the methods of some Die Brücke artists?

a. Having models from their social circle sit for quarter-hour poses to encourage spontaneity.

b. Having trained models sit for long stretches of time

c. Painting natural scenery as accurately as possible from memory.

d. Painting outdoors in natural light

6. Wassily Kadinsky (1866–1944) helped form the Blaue Reiter group. Which of the following best describes his artistic outlook?

a. All forms of art are equally capable of reaching a level of spirituality and rising above the materialism of society.

b. All forms of art stem from the same desire to subvert authority or strict structure.

c. Art that accurately depicts reality can stir up emotional reactions that encourage engagement with one's surroundings.

d. Art must be highly connected to the origins of humanity in order to evoke emotion within the viewer.

7. Wilhelm Heinrich Otto Dix (1891–1969) was one of the most famous members of the Neue Sachlichkeit. Which of the following best describes how his life experience aligned with that school of art?

a. A religious retreat wherein he had a spiritual awakening regarding the futility of pursuing material wealth.

b. Years of labor in manufacturing plants, wherein he witnessed wealthy business owners' exploitation of workers.

c. Traumatic experience in the Great War, including a recurring nightmare of crawling through destroyed houses.

d. Dissatisfaction with the highly idealized style of portraiture that was displayed in the Musee du Luxembourg in Paris.

8. Giorgio de Chirico was a metaphysical painter. Which of the following likely describes one of his metaphysical works, The Disquieting Muses (1916)?

a. Chirico uses imagery of mannequins set in a claustrophobic space, evoking a sense of irony and enigma and distorting perspective.

b. Chirico depicts a naturalistic scene with a pair of humanistic forms using pastel colors, evoking a sense of purity and closeness with nature.

c. Chirico uses a two-color palette to create a futuristic scene and evoke a sense of hopefulness and anticipation in his audience.

d. Chirico evokes the ethereal beauty of the mythical nine muses using soft and harmonious colors and forms.

9. Why is sculpture often described as the most Apollonian art form?

a. Sculpture appeals to the symbolic value of movement and chaos

b. Sculpture appeals directly to man's instinctive, chaotic emotions and not to his formally reasoning mind

c. Sculpture relies entirely on form and structure for its effect

d. Sculpture balances highly structured and free flowing elements to create different effects

10. Jane Schmidt's painting Color Field is described as using Dionysian elements. Which of the following descriptions would best align with such a characterization?

a. The frenzied strokes and lack of clear forms create a sense of passion and movement.

b. The clearly outlined structure of the forms evoke a sense of order and peace.

c. The bold strokes and overall tone of blue hues evoke a quiet, suppressed imbalance.

d. The use of lines, closed form, and dark tonalism create an impression of stillness and structure.
