# Supplementary material for: Testing the testing effect on prolific: when retrieval practice fails to boost learning
Source: Front Psychol. 2026 Jan 29;17:1727423. doi: 10.3389/fpsyg.2026.1727423 (PMC12894256; doi:10.3389/fpsyg.2026.1727423)
Supplement: Supplementary file 2 [file Presentation_2.pdf]

Lightning formation: Key ideas

1. Cool air moves.
2. It becomes heated.
3. It rises.
4. Water condenses.
5. The cloud extends beyond the freezing level.
6. Crystals form.
7. Water and crystals fall.
8. It produces updrafts and downdrafts.
9. People feel the gusts of cool wind before the rain.
10. Electrical charges build.
11. Negative charges fall to the bottom of the cloud (or positive charges go to the top).
12. A step leader travels down...
13. ...in a step fashion
14. The leaders meet...
15. ...at 165 feet from the ground
16. Negative charges rush down.
17. They produce a light that is not very bright.
18. Positive charges rush up.
19. This produces the bright light people see as a flash of lightning.

Meteorological knowledge questionnaire

Please place a check mark next to the items that apply to you.

- I regularly read the weather maps.
- I know what a cold front is.
- I can distinguish between cumulous and nimbus clouds.
- I know what a low pressure system is.
- I can explain what makes the wind blow.
- I know what the following symbol means.

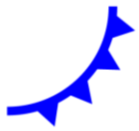

- I know what the following symbol means.

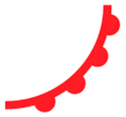

Rate your knowledge of weather.

Very Little / Little / Average / Much / Very Much
